# Rapid and Nondestructive Measurement of Rice Seed Vitality of Different Years Using Near-Infrared Hyperspectral Imaging

**DOI:** 10.3390/molecules24122227

**Published:** 2019-06-14

**Authors:** Xiantao He, Xuping Feng, Dawei Sun, Fei Liu, Yidan Bao, Yong He

**Affiliations:** 1 College of Biosystems Engineering and Food Science, Zhejiang University, Hangzhou 310058, China; hxt@zju.edu.cn (X.H.); pimmmx@163.com (X.F.); DZS0015@zju.edu.cn (D.S.); fliu@zju.edu.cn (F.L.); ydbao@zju.edu.cn (Y.B.); 2 Key Laboratory of Spectroscopy Sensing, Ministry of Agriculture and Rural Affairs, Zhejiang University, Hangzhou 310058, China

**Keywords:** seeds vitality, rice seeds, near-infrared spectroscopy, hyperspectral image, discriminant analysis

## Abstract

Seed vitality is one of the primary determinants of high yield that directly affects the performance of seedling emergence and plant growth. However, seed vitality may be lost during storage because of unfavorable conditions, such as high moisture content and temperatures. It is therefore vital for seed companies as well as farmers to test and determine seed vitality to avoid losses of any kind before sowing. In this study, near-infrared hyperspectral imaging (NIR-HSI) combined with multiple data preprocessing methods and classification models was applied to identify the vitality of rice seeds. A total of 2400 seeds of three different years: 2015, 2016 and 2017, were evaluated. The experimental results show that the NIR-HSI technique has great potential for identifying vitality and vigor of rice seeds. When detecting the seed vitality of the three different years, the extreme learning machine model with Savitzky–Golay preprocessing could achieve a high classification accuracy of 93.67% by spectral data from only eight wavebands (992, 1012, 1119, 1167, 1305, 1402, 1629 and 1649 nm), which could be developed for a fast and cost-effective seed-sorting system for industrial online application. When identifying non-viable seeds from viable seeds of different years, the least squares support vector machine model coupled with raw data and selected wavelengths of 968, 988, 1204, 1301, 1409, 1463, 1629, 1646 and 1659 nm achieved better classification performance (94.38% accuracy), and could be adopted as an optimal combination to identify non-viable seeds from viable seeds.

## 1. Introduction

Rice (*Oryza sativa* L.) is one of the three most important crops in the world, with a harvested area of 167 million ha and 769 million tons of total yield in 2017 [1]. However, the world population is increasing rapidly, and the total population will grow up to nearly 7.7 billion in 2019, compared with 6.9 billion in 2010, which will affect food security greatly and may lead to a food crisis around the world [2]. Numerous efforts have been made to satisfy this demand, such as optimizing the agronomic process, improving post-harvest technologies and biotechnology improvements in seeds and breeding mechanisms [3]. As an optimization means of agronomic processes, ensuring seed vitality and vigor is one of the most effective methods to increase crop production, which is particularly important for direct seeding, as it can not only enhance crop establishment but also increase the plant’s ability to compete against weeds [4].

Seed vitality and vigor directly affect the performance of seedling emergence and stand establishment [5]. Usually, any physical or biochemical damage to seeds can cause reduced or complete loss of vitality. More specifically, any changes in field conditions (e.g., humidity, temperature, pests, diseases) and post-harvest processes (e.g., drying, storage) can lead to seed damage, and thus cause retardation or complete vitality loss if not carefully controlled. These factors are, however, difficult to control. Therefore, the knowledge of whether a seed is viable or not before sowing is important both to seed companies and farmers. For seed companies, knowing seed vitality in advance helps them to determine the quality of their products, while for farmers it plays an important role in yield increase and prediction [6]. Determination of seed vitality is therefore necessary, and relevant studies should be conducted to build such a detection system for seed vitality.

Traditional detection methods of seed vitality, such as immunoassay tests, polymerase chain reaction tests and germination tests, could obtain the seed vigor intuitively, but they are expensive, time-consuming and destructive, which results in their low application in seed vigor detection [3]. Many research works have been conducted to construct potential rapid and non-destructive methods to measure seed vigor. Four non-destructive approaches with different techniques or principles, i.e., nuclear magnetic resonance spectroscopy [7], X-ray [8,9], laser speckle technique [10] and the measuring technology of seed conductivity [11] were investigated, however, they have not been widely used because of the low efficiency and complicated operation. Fortunately, recent studies show that molecular spectroscopic techniques, such as point-based and image-based hyperspectral techniques, have great potentials in the detection of seed ingredients with the advantages of high detection speed, non-destructive nature and low cost [12].

Point-based spectroscopic techniques, such as Raman, mid infrared, and Fourier transform-near infrared spectroscopies, acquire chemical information in a fixed-point area of the sample, and provide a large number of spectral details, but do not offer the spatial information that is important for seed detection application [13]. Hyperspectral imaging (HSI) is one of the most feasible methods for rapidly and non-destructively detecting the substances of agricultural products. It combines the technologies of spectroscopy and digital imaging, and is able to obtain spectral and spatial information simultaneously from testing samples in the form of a hypercube with two spatial dimensions and one spectral dimension [14]. Based on the spatial data, the HSI technique has the ability to collect hyperspectral information from samples of different sizes and shapes [15]. In addition, the detection speed of HSI is faster than that of point-based techniques, as many samples can be scanned and analyzed at the same time by using an HSI camera.

The HSI technique coupled with visible (vis) and/or near infrared (NIR) spectroscopy is generally used to identify or inspect different substances of seed by recognizing the molecular bonds in the sample. Many studies have been conducted to detect the vitality of seeds for different species. The corn with a large grain size and flat shape has been paid more attention for seed vitality detecting. Collins et al. measured corn seed vitality using short wave infrared line-scan hyperspectral imaging, and the results indicated that hyperspectral imaging can be used to accurately classify corn based on vitality [3]. Ashabahebwa et al. assessed the performance of testing corn seed vitality by applying the Fourier transform near-infrared spectroscope [16]. In addition, the detections of vitality and vigor for seeds of oat [17], muskmelon [18], soybean [19,20] and watermelon [21] were developed with the HSI technique. Previous studies have shown the potential of using HSI coupled with multivariate data analysis for the detection of internal conditions of rice seeds, such as origin [22], variety [23,24,25], nitrogen content [26], moisture content [27] and heavy metal concentration [28]. To the best of our knowledge, many studies were conducted only for vitality detection of artificially aged seed, and, so far, no study has been carried out to detect the vitality of rice seeds under natural ageing conditions by using HSI, even though the results obtained from natural ageing seeds were more consistent with the actual situation.

This study was conducted to determine the optimal spectral wavebands and multivariable classification model to acquire or detect the vigor of rice seeds stored for different years based on the near-infrared hyperspectral imaging (NIR-HSI) technique, and attempt to build a model to identify non-viable seeds from viable seeds of different years, and ultimately provide an alternative approach of rapidly and non-destructively measuring the rice seed vitality for industrial or large-scale application.

## 2. Results and Discussion

### 2.1. Spectral Interpretation

**Figure 1:**
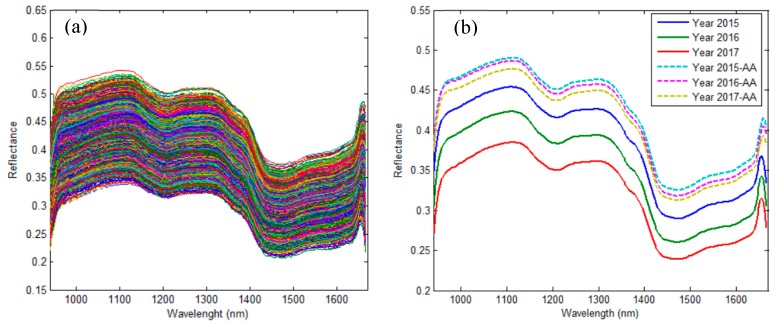
(**a**) Raw spectra of all rice simples and (**b**) mean spectra for rice seeds.

A raw spectral data plot and mean raw spectral data plot from selected regions of interest (ROI) are shown in Figure 1a,b, respectively. The change trends of the spectral reflectance curves of all rice kernels showed clear similarities. As shown in Figure 1b, the seed spectral curves of three different years had large differences in the reflectance of wavebands, while the differences were negligible after all three year seeds were artificially aged to lose vigor. The germination tests on the representative samples showed a high vitality, with a germination rate of 95%, 92.86% and 80.71%, and vitality index of 261.26, 225.6 and 154.15 for rice seeds of the years 2017, 2016, and 2015, respectively, as shown in Table 1. It is obvious that the germination rate and vitality index reduced as the year of preservation increased, which was consistent with the spectral change of rice seeds, and could be used as a basic principle for classifying rice seeds of different years. All germination rate values were higher than the factory labelled 80% germination rate, indicating the seeds stored within three years still have enough vitality to be used in rice production. The seeds that were subjected to microwave heat treatment were similar to the non-viable seeds, and their germination rate and vitality index were both tested to be zero, which resulted in a higher spectral reflectance of artificial aging seeds. Moreover, the spectral reflectance of aged seeds with non-vitality had high similarity, no matter the year of seeds. Therefore, it is difficult to identify the year of aged non-viable seeds using hyperspectral imaging; however, it is highly possible to identify non-viable seeds from common viable seeds of three different years.

### 2.2. The Results of Principal Component Analysis

**Figure 2:**
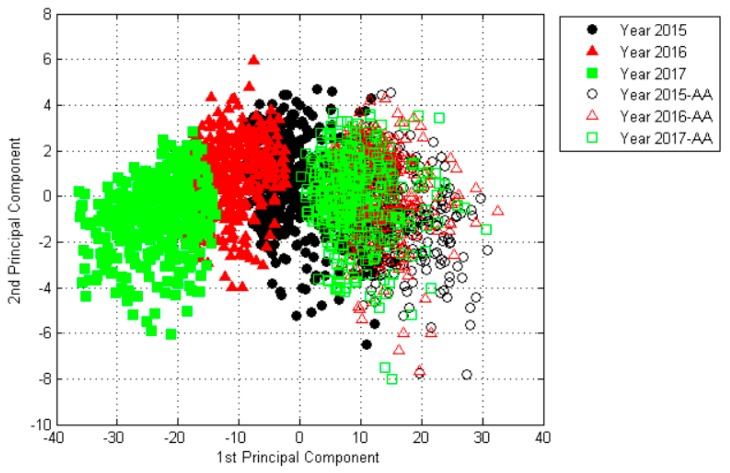
Principal component analysis (PCA) results for raw data based on the spectral data of all six seed groups. AA: artificial ageing.

Principal component analysis (PCA) is one of the most popular multivariate statistical techniques in almost all scientific disciplines. It uses an orthogonal transformation to convert a set of observations of possibly correlated variables into a set of values of linearly uncorrelated variables called principal components [29]. PCA was used in the study for data exploration and classification feasibility analysis. Figure 2 shows PCA results for raw data based on the spectral data of all groups of seeds. The analysis of PCA results shows that the first two principal components (PCs) were found in up to 99.58% of all the variability—PC1 and PC2 had 97.94% and 1.64% variance, respectively. That is to say, these two PCs showed the most significant variation among samples, and could explain 99.58% of all the variability. As illustrated in Figure 2, the PCA data of non-viable seeds of three different years in this plane projection were more concentrated, while an obvious difference occurred for the viable seeds of three different years. As a result, the viable seeds of different years were more likely to be classified with each other, while the seeds were difficult to differentiate after the three kinds of seeds were artificially aged to lose vigor because of the high overlap between the groups shown in Figure 2.

**Figure 3:**
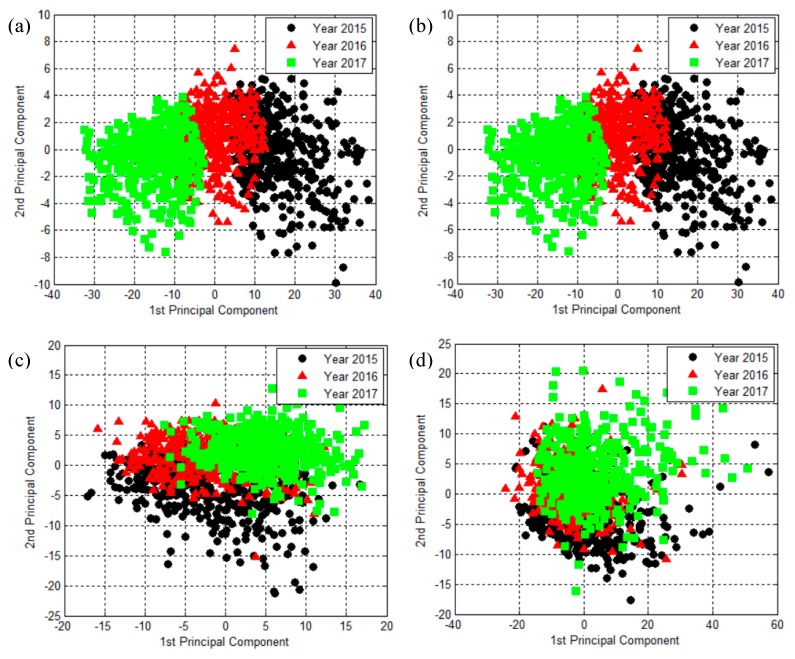
PCA results for (**a**) raw data and preprocessed data of (**b**) Savitzky–Golay (SG), (**c**) Savitzky–Golay first derivative (SG-D1) and (**d**) multiplicative scatter correction (MSC), based on the spectral data of rice seeds of different years.

The PCA technique was utilized to analyze the spectral data of viable seeds of three different years at the three different preprocessing methods, and the results are illustrated in Figure 3. The PCA results showed that differences among three samples have better data clustering performance using Savitzky–Golay (SG) preprocessing algorithms compared with other models (Figure 3b). However, PCA results for Savitzky–Golay first derivative (SG-D1) and multiplicative scatter correction (MSC) showed preprocessed data generated much less distinctive clustering results (Figure 3c,d), which was worse than the raw data (Figure 3a). It may have been that the noise was overamplified when spectral data was preprocessed by SG-D1 and MSC methods, thus resulting in a lower signal-to-noise ratio and less distinctive clustering for the three groups. The raw data obtained a better clustering performance due to the data being calculated and obtained based on the mean spectral data of the region of one rice seed, which could remove spectral noises in the seed to some extent.

### 2.3. Optimal Wavelengths Selection

A classification model established by applying a number of highly correlated variables would increase the computational complexity for predicting. Thus, selecting important and irrelevant wavelengths from hyperspectral data is necessary before establishing the discriminant model. In this study, the successive projections algorithm (SPA) was proposed to determine the optimal wavelengths for predicting rice seed vitality based on SG, SG-D1 and MSC preprocessed data and the raw data. The numbers of wavelengths selected by SPA were decreased to 4.2, 3.7, 5.1 and 2.8% of all 216 wavelengths. Then, the selected wavelengths were used to build multivariate classification models for the determination of rice vigor, including the partial least square-discriminant analysis (PLS-DA), the least squares support vector machines (LS-SVM) and the extreme learning machine (ELM).

**Figure 4:**
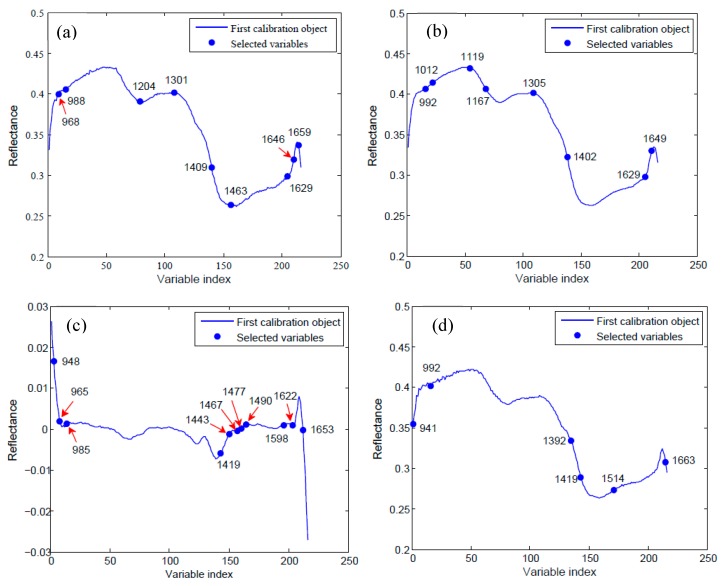
Selection of optimal wavelengths by successive projections algorithm (SPA). Distributions of important variables (marked with ‘filled circle’) for (**a**) raw data and preprocessed data of (**b**) SG, (**c**) SG-D1 and (**d**) MSC.

In general, spectral absorptions at the optimum wavelengths had a notable correlation with the molecular structures of chemical components. Some important wavelengths (988, 1409, 1629 and 1659 nm) were shared by data of raw, SG, SG-D1 and MSC (Figure 4), and may have been responsible for the germination ability of the rice seed. The absorption band near 988 nm may be assigned to the second overtone of the O–H vibration bond overtone of water [18,30]. The wavelength band near 1409 was primarily attributed to the O–H first overtone, which are common in starch and lipids [31]. The wavelengths near 1629 and/or 1659 nm were assigned to the first overtone of O–H stretching, C–H from the methylene group and the N–H stretch first overtone, which refer to the CONH representing the protein content [31]. Lipid peroxidation, loss of protein function and hydrolysis of starch have been suggested as causes for loss of seed vitality [32]. Thus, the selected wavelengths related to starch, lipids and protein structures were the foundation for discrimination between the three groups. In addition, wavelengths selected from SG preprocessed data had roughly the same distribution as that of raw data, and the common wavebands of 1204 nm and 1301 nm were connected to the second overtone of C–H harmonic stretching [33]. As for the SG-D1 and MSC preprocessing methods, most wavelengths were located in the range of 1392–1514 nm, which mainly corresponded to the first overtone of C–H stretching and deformation of CH_2_ and CH_3_ groups [33].

### 2.4. Classification Model Results

After optimal wavelength selection, the whole spectral data set was reduced to a matrix of dimensions m × n, where m represents the number of samples (m = 2400) and n was the number of selected wavelengths including 9, 8, 11 and 6 for raw data, SG, SG-D1 and MSC preprocessed data, respectively. To determine the suitability of optimal variables selected by SPA, the optimal wavelengths were used to build multivariate models, including PLS-DA, LS-SVM and ELM for classifying the samples.

#### 2.4.1. Assessment of Seed Vitality of Three Different Years

**Figure 5:**
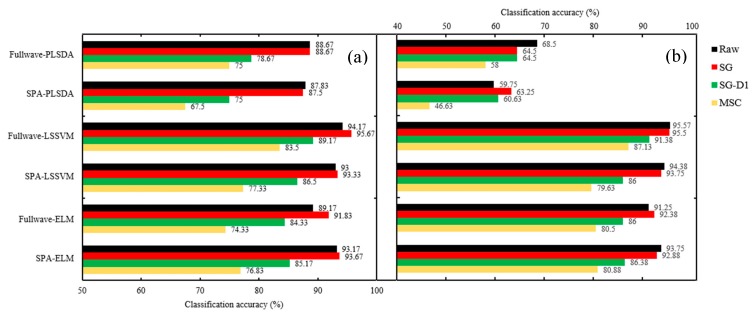
The prediction results of classification models for identifying (**a**) seed vitality of three different years and (**b**) non-viable seeds from viable seeds of three different seeds.

The seed vitality was different in the seeds of three different years, and the seeds stored in later years could obtain a higher vigor, which was consistent with the change trend of spectral reflectance of the seeds (i.e., the reflectance of seeds stored in earlier years was generally higher than that of later years). Based on this principle, three models of PLS-DA, LS-SVM and ELM were built to identify the vitality of seed samples of different years. The classification accuracy of calibration set varied from 64.67% to 97.5%, and the accuracy range of prediction set was 67.5–95.67%. The lowest 64.67% accuracy of the calibration set and 67.5% accuracy of the prediction set were obtained by using the PLS-DA model with MSC preprocessing and the SPA method, while the highest values of 97.5% and 95.67% for the calibration and prediction set, respectively, were achieved when using the LS-SVM mode with SG preprocessing and full-wave bands. As for the classification results of the prediction set (Figure 5), the PLS-DA model with selected wavelengths had the lowest classification accuracy in the three classification models (87.83, 87.5, 75 and 67.5% for raw, SG, SG-D1 and MSC, respectively). Applying preprocessing and wavelength selection methods before model application had no improvement in classification accuracy. The LS-SVM model gave the highest accuracy of the three models with/without data preprocessing procedures—with up to 95.67% accuracy using the data of SG preprocessing in the full-wave bands—and could reach the high accuracy of 93.33% by applying the reduced wavelengths selected by SPA. The good performance of the LS-SVM model is probably because its decision boundaries can become much clearer after transforming the data into higher dimensions, and as a result it classified different groups more accurately. However, the PLS-DA models establish decision boundaries based on the thresholds under low dimensions, and thus this results in misclassifications due to outliers [34]. The ELM model, a simple tuning-free three-step algorithm with a fast learning speed, achieved a result of accuracy of 93.67% based on the reduced wavelengths of SPA, along with SG preprocessing, which was even a little higher than the 93.33% accuracy of LS-SVM under the same condition. Though the accuracy of 93.67% was lower than the 95.67% accuracy of the LS-SVM model with SG preprocessed data in the full-wave bands, its data processing load with only eight wave bands (992, 1012, 1119, 1167, 1305, 1402, 1629 and 1649 nm) decreased to 3.7% of the classification model of full wavelengths, which is a significant performance improvement for an almost 27-fold increase in data processing speed. Therefore, the ELM model coupled with the variable-selection method of SPA and the preprocessing method of SG could be adopted as an optimal combination to classify the seed of different years for a fast and cost-effective seed-sorting system for industrial online application.

#### 2.4.2. Identifying Non-Viable Seeds from Viable Seeds of Different Years.

The seed samples, no matter whether they were stored in year 2015, 2016 or 2017, all lost vitality completely, with a germination rate of 0% and vitality index of 0 after they underwent artificial aging. The spectral reflectance of aged seeds increased greatly and differed from that of the seeds of three years (Figure 1b), which provides a possibility to pick out non-viable seeds from normal viable seeds stored in different years. Furthermore, 133, 133 and 134 seeds were selected randomly from aged seeds of the years 2015, 2016 and 2017, respectively. In total, 400 aged seeds were obtained and then used as a non-viable group with other three viable groups of different years (i.e., 2015, 2016 and 2017) to build classification models for evaluating the performance of identifying non-viable seeds. The results are shown in Table 2. The classification accuracy of the calibration set varied from 48.75% to 96.38% and the accuracy range of the prediction set was 46.63–95.57%. The classification accuracy of the calibration set was generally higher than the accuracy of the prediction set at the same conditions. As for the classification results of the prediction set (Figure 5b), PLS-DA with less than 70% accuracy was the model of lowest classification accuracy, which was even lower than the accuracy of the PLS-DA model used for classifying seed vitality of merely three different years. The LS-SVM model gave the highest accuracy of the three models with/without data preprocessing procedures, with up to 95.57% accuracy using the raw data in the full-wave bands, and could reach the high accuracy of 94.38%, applying the reduced wavelengths selected by SPA. The ELM model achieved a result of accuracy of 93.75% based on the reduced wavelengths of SPA with raw data, which was slightly lower than the accuracy of 94.38% of LS-SVM under the same condition. Though the accuracy of 94.38% of the LS-SVM model was lower than the 95.57% accuracy of LS-SVM with raw data in the full-wave bands, its data processing load with nine wave bands (968, 988, 1204, 1301, 1409, 1463, 1629, 1646 and 1659 nm) decreased to 4.2% of the classification model of full wavelengths, which is a significant performance improvement for an almost 23.8-fold increase in data processing speed. Therefore, the LS-SVM model coupled with the variable-selection method of SPA and raw data could be adopted as an optimal combination to identify non-viable seeds from viable seeds.

## 3. Materials and Methods

### 3.1. Samples and Sample Preparation

In this study, the rice seeds of ShenLiangYou862 from three different years, including 2015, 2016 and 2017, were selected to be investigated, which were kindly provided by a commercial company (Jiangsu Tomorrow Seed Technology LLC, Nanjing, China). The seeds were cleaned first, and damaged seeds were removed. When acquiring hyperspectral images, three samples in different years could not be differentiated by the naked eye. For each category, 800 kernels were acquired with 400 seeds used as the different-year sample and the other 400 seeds used as the aged sample for comparison. Artificial aging of seeds was induced in the rice simples using microwave heat treatment at 700 W input power and 60 s exposure time, which was optimized in advance for this experiment in accordance with the study by Ambrose et al. [35]. 

### 3.2. Hyperspectral Image Collection

**Figure 6:**
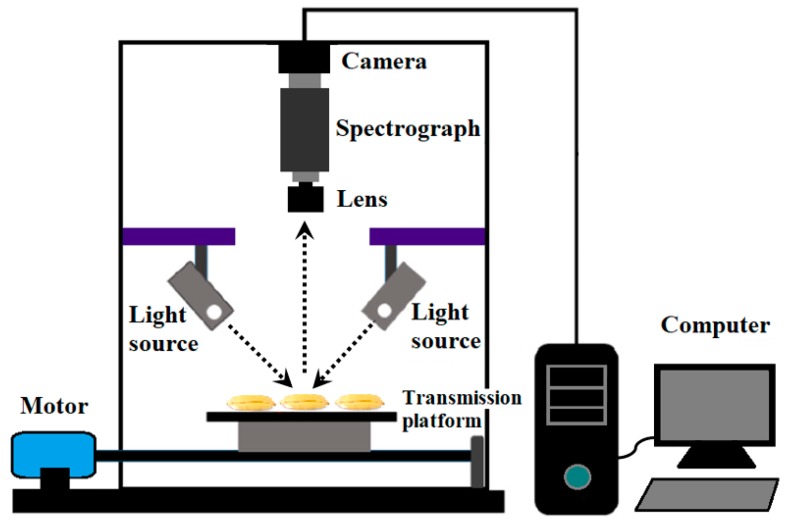
Schematic of line-scan near-infrared hyperspectral imaging (NIR-HSI) system and scanning of seed samples.

**Figure 7:**
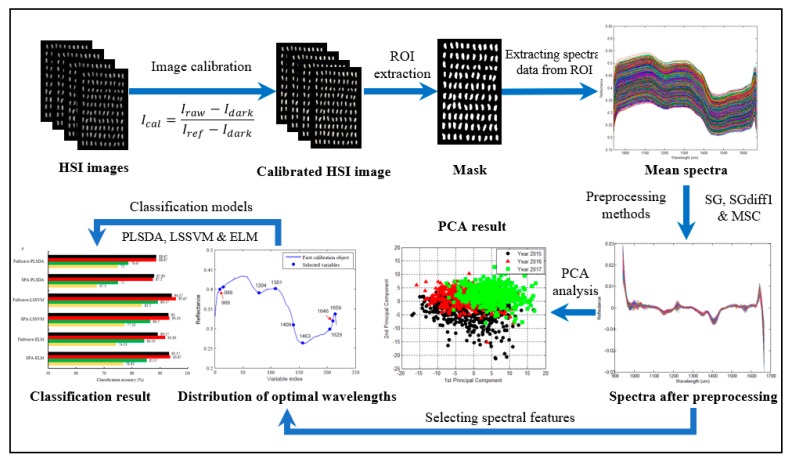
Schematic overview of the analytical procedure for identifying the vitality of different years. ROI: regions of interest.

A line-scan NIR-HSI system was used to acquire the hyperspectral images of rice seeds, as shown in Figure 6. The system comprised an imaging spectrograph (ImSpector N17E; Spectral Imaging Ltd., Oulu, Finland) that covered the spectral range of 874–1734 nm with a spectral resolution at 3.36 nm, a charge coupled device camera (Xeva 992; Xenics Infrared Solutions, Leuven, Belgium) with the spatial resolution of 320 × 256 pixels, two line light sources (Fiber-Lite DC950, Dolan Jenner Industries Inc., Boxborough, MA, USA), a transmission platform (IRCP0076, Isuzu Optics Crop, Taiwan), a dark box and a computer. In order to acquire clear and non-deformable hyperspectral images, the moving speed of the transmission platform, the exposure time and the work distance between samples and the camera were adjusted to 19 mm/s, 3.5 ms and 23.4 cm, respectively. Rice seeds were placed on a dark-background sampling plate irrespective of whether the germinal side of the kernel was facing the camera, then the sampling plate was transferred to the transmission platform for scanning seeds line by line. Spatial and spectral data were obtained from the sample when it was moved into the range of the camera filed. After scanning the samples for hyperspectral data, the hyperspectral images were calibrated by the following equation:

(1) $$I_{\mathrm{cal}}=\left(I_{\mathrm{ra}w}-I_{\mathrm{dark}}\right)/\left(I_{\mathrm{ref}}-I_{\mathrm{dark}}\right),$$

where *I_cal_*, *I_raw_*, *I_dark_* and *I_ref_* are the corrected images, original images, dark current and reference images, respectively. *I_ref_* was measured using a white Teflon tile with the reflectance close to 99%, and *I_dark_* was collected by covering the camera lens completely with the cap provided by the manufacturer. The calibrated HSI image was ultimately obtained to analyze the spectral data in every single seed (Figure 7). Spectral data before 941 nm and after 1666 nm were omitted because of low signal-to-noise ratio, which was mainly caused by bad pixels on the camera detector, lighting characteristics and the movement of the transmission platform.

### 3.3. Data Extraction and Preprocessing

A threshold value of 0.15 was used to segment calibrated hyperspectral images to remove the effect of the background and to obtain only seed pixels. The regions of interest (ROI) were selected by applying the 1301 nm band image, and then spectral information of the respective rice sample in the HSI images was extracted relying on the ROI. The spectra of each pixel in the ROI were averaged for each seed, and, in total, 2400 average spectra representing 2400 scanned seeds were calculated and saved for further analysis.

Three preprocessing methods were used in this paper to correct the spectral data, including the Savitzky–Golay smoothing (SG), the Savitzky–Golay first derivative (SG-D1) and multiplicative scatter correction (MSC).

The SG method is a digital filter that can be applied to a set of digital data points for the purpose of smoothing the data, which can effectively keep useful information and reduce high-frequency noise in a hyperspectral image. The polynomial order and number of points in the SG method are two computation parameters, which were adjusted to 3 and 15, respectively, for a good effect in spectrum smoothness.

The SG-D1 method is the first derivative form of the SG method. By deriving SG data, it has the advantages of emphasizing the spectral features of the data and removing the additive baseline; however, it inevitably amplifies the noise at the same time, which may have a large impact on the classification results. The polynomial order and number of points was also set to 3 and 15, respectively, when the SG process was executed.

The MSC method was used to remove physical effects, such as particle size and surface blaze, from the spectra, which do not carry any chemical or physical information. This method is capable of correcting differences in the baseline and has an advantage of the transformed spectra being similar to the original spectra, and optical interpretation is therefore more easily accessible [36].

### 3.4. Spectral Feature Selection

Hyperspectral images could provide a large amount of spectral and spatial information related to the vitality properties of the rice seeds; nevertheless, they also contain overlapping and redundant information. It is necessary to apply a feature selection algorithm to obtain representative and important wavelengths for reducing irrelevant information and improving computation speed. 

The successive projections algorithm (SPA) is a variable-selection technique that has attracted increasing interest in the analytical-chemistry community in the past 10 years. In SPA, the selection of variables is cast in the form of a combinatorial optimization problem with constraints, and projection operations in a vector space are used to choose subsets of variables with a small degree of multi-collinearity in order to minimize redundancy and ill-conditioning problems [37]. The algorithm SPA was applied in this study to select the optimal wavelengths. The selected wavelengths with the minimum collinearity have the maximum projection value on the orthogonal subspace.

### 3.5. Construction and Analysis of Classification Models

In this paper, three discriminant models were built and analyzed, including the partial least square-discriminant analysis (PLS-DA), the least squares support vector machine (LS-SVM) and the extreme learning machine (ELM).

The partial least squares (PLS) algorithm was first induced for regression tasks and then evolved into a classification method that is well known as PLS-DA. This method is a popular chemometrics technique used to optimize the separation between different groups of samples, which is accomplished by linking raw data and class membership [38], as described in Equation (2):

(2) Y=X·B+F,

where Y is the n × 1 vector of the response variables that relates to the measured sample categories, B is the regression coefficients matrix for the spectral variables, F is the n × 1 error vector of residuals, X is the n × j data matrix of the spectral variables for each measured sample category, n is the number of samples and j is the number of variables. During the model development and updating stages, the number of main components was optimized by 10-fold cross validation and ultimately 10 main components were determined.

Known as the least square form of the support vector machine (SVM) approach, LS-SVM applies an equality constraint instead of an inequality constraint that has been used in SVM to obtain a linear set of equations. As a result, it simplifies the complex calculation and is easy to train. It has been reported that the LS-SVM could present a remarkable performance, as it maps the data input space into a high-dimensional feature space through a kernel function (the radial basis function (RBF) kernel function was applied in this paper). The two main parameters of the SVM method, including the penalty factor and the radial width of the kernel function, are optimized using a grid-search algorithm coupled with 10-fold cross validation during the model development and updating stages [39].

ELM has shown the advantages of fast learning speed and excellent generalization performance compared to traditional feedforward network learning algorithms such as back-propagation (BP). In most cases, ELM is used as a simple learning algorithm for single-hidden layer feedforward neural network (SLFN). Due to its different learning algorithm implementations for regression, classification or clustering, ELM has also been used to form multi hidden layer networks, deep learning or hierarchical networks [40]. The hidden node in ELM is a computational element, which is considered as a classical neuron, and its number was tuned to 100 for high accuracy. 

Based on the spectral data with different preprocessing methods—i.e., SG, SG-D1 and MSC—the performances of the three models above were analyzed and evaluated to classify the vitality of seeds stored for different years. For 400 samples of each category, 200 seeds were used as the training sample and the other 200 seeds were used as the testing sample.

### 3.6. Germination Test

After hyperspectral images of all seeds were collected, 140 seeds were randomly selected from each group for the germination test following the International Seed Testing Association (ISTA) guidelines [41]. Seeds for germination were placed between two wet germination papers and incubated in a germination chamber for 7 days. The germination chamber was set as day-night mode at 30 °C, 80% RH and 10,000 Lx during the day (16 h), and 20 °C, 80% RH and 0 Lx during the night (8 h). Germination results of seeds were recorded daily and seeds with a 1 cm germ length were counted as germinated according to ISTA standards. The germination rate (GR, %) was calculated by Equation (3). The seeds high in vigor generally provided early and uniform stands, indicating that the seeds had the potential to produce vigorous seedlings under favorable conditions. Therefore, in this study, germination days were considered as a standard for seed vigor, and used as a factor to determine the vitality index (VI), as shown in Equation (4):

(3) GR=GN/SN·100%,

(4) $$VI=S\cdot \sum \frac{Gt}{Dt},$$

where GN and SN are the numbers of germinated and non-germinated rice seeds, respectively, which were recorded on the last day of the germination test, S is the average value of germ length (cm), Dt is the number of the day t and Gt is the germination number recorded on the day of Dt.

## 4. Conclusions

The NIR-HSI technique, combined with multiple preprocessing methods and classification models, was used to identify the vitality of rice seeds. Spectral data was extracted from the ROI of the hyperspectral image and three preprocessing methods, including SG, SG-D1 and MSC, were applied to reduce the effect of irregularities in the spectral data caused by factors such as random noise, light scattering and sample texture. The SPA algorithm was adopted to obtain optimal wavelengths for the vitality of seeds, and to reduce computational cost. The numbers of selected wavelengths were 9, 8, 11 and 6 for raw data, SG, SG-D1 and MSC preprocessed data, respectively, which could decrease data processing load greatly compared to the classification model of full wavelengths. Then, these optimal wavelengths, as well as full wavelengths, were used to build multivariate models, including PLS-DA, LS-SVM and ELM, for determinate seed vitality of three different years and non-viable seeds from viable seeds of three different seeds. As for the detection of seed vitality of the three different years, better performance could be achieved by using pretreatment SG compared with the other two preprocessing methods. The classification accuracies for the seed vitality of three different years obtained using PLS-DA, LS-SVM and ELM with selected wavelengths and SG preprocessing were 87.5%, 93.33% and 93.67%, respectively. The ELM-SG method with spectral data from only eight wavebands (992, 1012, 1119, 1167, 1305, 1402, 1629 and 1649 nm) had better and faster classification performance, and could be developed to a fast and cost-effective seed-sorting system for industrial online application. As for identifying non-viable seeds from viable seeds of different years, the LS-SVM model coupled with raw data and selected wavelengths of 968, 988, 1204, 1301, 1409, 1463, 1629, 1646 and 1659 nm, achieved a classification accuracy of 94.38%, which decreased the data processing load to 4.2% of the classification model of full wavelengths and could be adopted as an optimal combination to identify non-viable seeds from viable seeds.

## Figures and Tables

**Table 1:** Germination rate and vitality index of all sets of seeds as determined by germination test.

Years of Seed	Treatment	Germination Number	Non-Germination Number	Germination Rate (GR)	Vitality Index (VI)
2015	−	113	27	80.71%	154.15
	AA	0	140	0	0
2016	−	130	10	92.86%	225.6
	AA	0	140	0	0
2017	−	133	7	95%	261.26
	AA	0	140	0	0

AA: artificial ageing.

**Table 2:** The results of classification models established by full and selected wavelengths with different preprocessing methods.

		IVY						INV					
		PLS-DA		LS-SVM		ELM		PLS-DA		LS-SVM		ELM	
		Full.	Sel.	Full.	Sel.	Full.	Sel.	Full.	Sel.	Full.	Sel.	Full.	Sel.
Raw	Cal.	92.17	86.83	96.67	95.83	95.5	93.5	69.75	58.38	96	95.13	94.75	94.13
	Pre.	88.67	87.83	94.17	93	89.17	93.17	68.5	59.75	95.57	94.38	91.25	93.75
SG	Cal.	87.75	87	97.5	94.33	95.67	94.17	62.63	62.13	96.38	93.5	95.25	93.13
	Pre.	88.67	87.5	95.67	93.33	91.83	93.67	64.5	63.25	95.5	93.75	92.38	92.88
SG-D1	Cal.	79.17	73.67	94.67	86.17	90.17	85.5	66.25	61.13	95.75	87.13	91	86.38
	Pre.	78.67	75	89.17	86.5	84.33	85.17	64.5	60.63	91.38	86	86	86.38
MSC	Cal.	78.83	64.67	87.33	78	82.83	79	61.25	48.75	94.25	77.88	86.25	80.63
	Pre.	75	67.5	83.5	77.33	74.33	76.83	58	46.63	87.13	79.63	80.5	80.88

Cal.: calibration; Pre.: prediction; Raw: raw data; IVY: identification of the seed vitality of three different years; INV: identifying non-viable seeds from viable seeds; Full.: full wavelengths; Sel.: selected wavelengths by SPA; PLS-DA: partial least square-discriminant analysis; LS-SVM: least squares support vector machines; ELM: extreme learning machine.
